# Use of rituximab in SARS-CoV-2-positive renal transplant recipient with EBV reactivation and probable haemophagocytic lymphohistiocytosis

**DOI:** 10.1007/s13730-022-00711-4

**Published:** 2022-06-22

**Authors:** Derek Chan, Sabina Karimi, George Follows, Nicholas Torpey, Ondrej Suchanek

**Affiliations:** 1 Department of Clinical Nephrology and Transplantation, Addenbrooke’s Hospital, Cambridge University Hospitals NHS Foundation Trust, Cambridge, UK; 2 Department of Haematology, Addenbrooke’s Hospital, Cambridge University Hospitals NHS Trust, Cambridge, UK

**Keywords:** COVID-19, Renal transplant, EBV reactivation, Haemophagocytic lymphohistiocytosis, B cell, Rituximab

## Abstract

We present a case of a rapid clinical recovery in a critically ill kidney transplant recipient with SARS-CoV-2 positivity, Epstein–Barr virus (EBV) reactivation and probable secondary hemophagocytic lymphohistiocytosis (HLH) treated with etoposide-free regimen, based on dexamethasone and a single dose of rituximab. Although rituximab is often a part of EBV-HLH treatment strategy, its use in simultaneous Coronavirus 2019 disease (COVID-19) and solid-organ transplantation has not been reported yet. We review the current evidence for the potential of SARS-CoV-2 to trigger EBV reactivation, leading to a severe clinical illness. Finally, we compare the clinical features of hyper-inflammatory response typical for severe COVID-19 and classical secondary HLH and discuss the benefits of therapeutic B-cell depletion in both conditions.

## Presentation

A 33-year-old male renal transplant recipient presented to our Emergency Department (ED) in March 2020 (i.e. the beginning of the UK COVID-19 pandemic) with a 6-day history of intermittent fever, diffuse myalgia, arthralgia, non-productive cough and headache. On his arrival, he was pyrexial (39.1 °C) and tachycardic (115/min), with normal blood pressure (133/79 mmHg), respiratory rate (16/min) and oxygen saturations (99% in air). His physical examination was unremarkable except for bronchial breathing across his right hemithorax. He denied any dysuria, abdominal pain, diarrhoea, vomiting or neck stiffness.

His past medical history included Dent’s disease (an X-linked tubulopathy with nephrocalcinosis) leading to end-stage renal failure (ESRF) in 2004. In the same year, he received his first renal transplant from his father. Unfortunately, this allograft failed in 2008 secondary to recurrent episodes of hypovolaemia. He subsequently returned to peritoneal dialysis and underwent bilateral native nephrectomies. In 2011, he received a second living unrelated transplant with Human Leukocyte Antigen (HLA) mismatch in four out of six donor alleles (2 mismatches in HLA-A, 1 in HLA-B and 1 in HLA-DR locus); cytomegalovirus (CMV) donor positive/recipient negative and EBV donor positive/recipient positive. His transplantation and post-operative period was uncomplicated. Over the past nine years, he had been generally well with a stable allograft function (baseline creatinine 150 umol/L, eGFR 51 mL/min/1.73m2) on standard maintenance immunosuppression (tacrolimus 8 mg daily, mycophenolic acid 720 mg twice daily and prednisolone 5 mg daily). He works as an office worker but had been shielding himself at home in the week preceding the onset of his symptoms.

## Investigations and diagnosis

Initial blood tests revealed an acute inflammatory response with lymphopenia, as well as acute kidney injury (AKI) stage 1 and elevated tacrolimus level (Table 1). Admission chest X-ray (CXR) was normal. His renal graft ultrasound excluded any perfusion defect or obstruction. Our initial working diagnosis was COVID-19 with a mild kidney graft dysfunction most likely secondary to sepsis/dehydration and calcineurin inhibitor (CNI) toxicity. Other differentials included CMV/EBV viraemia or bacterial sepsis such as graft pyelonephritis.

**Table 1:** Laboratory investigations during the first half of hospital admission

Variable	Reference range	Day 1	Day 3	Day 6	Day 9*	Day 12
Haematology						
Haemoglobin (g/L)	135–172	102	99	101	85	78
White cells (× 10^9^/L)	3.9–10.2	4.7	6.4	6.9	8.3	9.7
Differential count (× 10^9^/L)						
Lymphocytes	1.1–4.5	0.49	0.45	0.77	1.78	3.30
Basophils	0–0.2	0.02	0.00	0.00	0.13	0.00
Eosinophils	0.02–0.5	0.24	0.32	0.74	0.27	0.19
Monocytes	0.1–0.9	0.42	0.96	0.49	0.56	0.29
Neutrophils	1.5–7.7	3.23	4.67	3.98	4.73	5.72
Platelets (× 10^9^/L)	150–370	141	115	144	116	151
Prothrombin time international normalised ratio	1–1.15	1.45	–	1.83	1.39	1.20
Fibrinogen (g/L)	1.46–3.33	–	2.71	2.45	2.12	1.11
Biochemistry						
Sodium (mmol/L)	133–146	130	129	126	136	137
Potassium (mmol/L)	3.5–5.3	4.2	4.8	5.5	4.9	4.7
Urea (mmol/L)	2.5–7.8	12.4	15.5	28.7	11.8	31.0
Creatinine (μmol/L)	62–115	202	311	594	137	250
Alanine transaminase (U/L)	10–49	43	60	105	122	94
Alkaline phosphatase (U/L)	30–130	127	223	428	910	717
Albumin (g/L)	35–50	32	27	22	17	23
Total bilirubin (μmol/L)	0–20	12	34	117	98	46
Tacrolimus (μg/L)	5–8	> 30	25.5	11.3	5.1	3.6
C-reactive protein (mg/L)	0–6	256	276	309	235	53
Procalcitonin (ng/mL)	0–0.5	6.13	–	–	13.25	12.59
Ferritin (μg/L)	22–322	–	3164.7	8937.9	8829.9	2640.0
Triglyceride (mmol/L)	0.3–1.8	–	1.61	1.74	1.82	3.82
Interleukin-1 beta (pg/mL)	0–3.1	–	–	2.04	0.10	–
Interleukin-6 (pg/mL)	0–2	–	–	434.31	8.82	–
Interleukin-10 (pg/mL)	0–1	–	–	20.99	14.59	–
Tumour necrosis factor alpha (pg/mL)	0–5	–	–	250.19	66.82	–
Interferon gamma (pg/mL)	0–10	–	–	52.84	7.81	–
Virology						
Nasopharyngeal SARS-CoV-2 (cycle threshold)	N/A	27	–	–	–	15
EBV PCR	N/A	1.3 × 10^7^	–	–	–	–
Urine analysis						
Blood	N/A	Neg	Trace	–	–	Trace
Leukocytes	N/A	Neg	Neg	–	–	Neg
Protein	N/A	–	3 +	–	–	Neg

*48 h post RTX administration and commencement of dexamethasone

## Management

On admission, tacrolimus and mycophenolic acid were withheld and his prednisolone dose doubled. He was re-hydrated with IV fluids and started on intravenous piperacillin–tazobactam empirically. Subsequently, his nasopharyngeal swab returned positive for SARS-CoV-2 by polymerase chain reaction (PCR). At the same time, his blood EBV PCR revealed a significant DNAemia (1.3 × 10^7^ copies/mL).

**Fig. 1:**
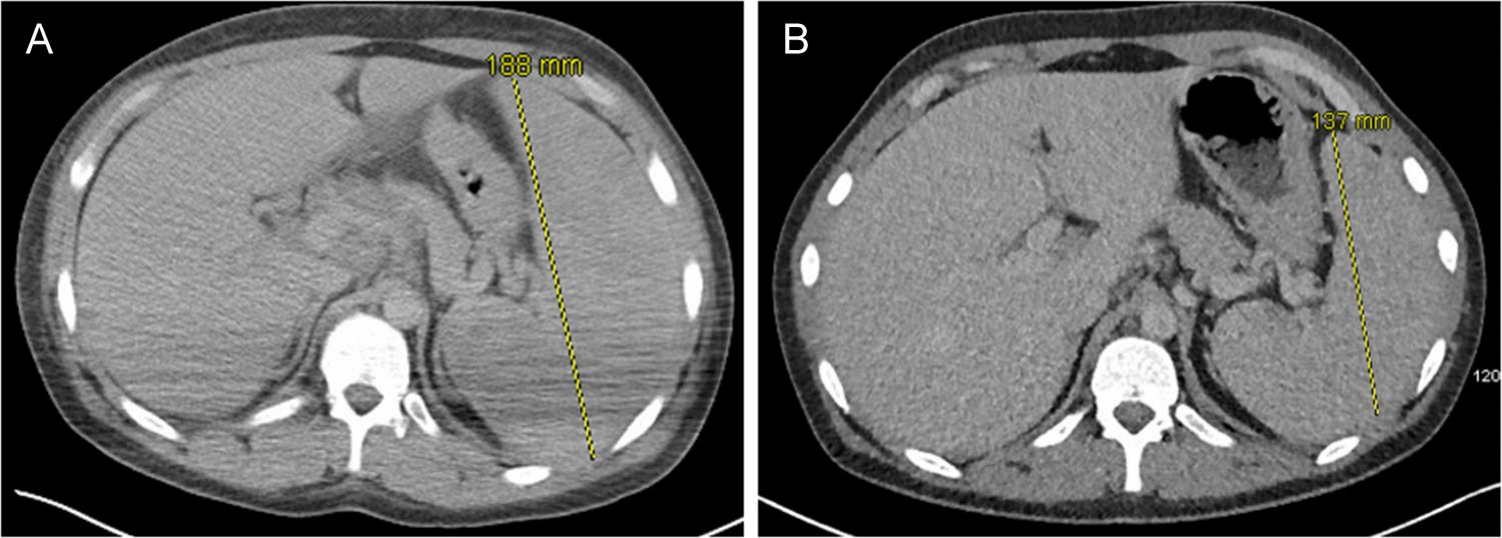
Axial CT images of the abdomen pre- (**A**) and 18 days post-treatment (**B**). Splenomegaly, with mediastinal, porta hepatis and para-aortic lymph node enlargement is demonstrated in **A** and its significant reduction in **B**. Splenic transverse diameter (yellow line) and corresponding measurement shown

The patient’s condition deteriorated shortly thereafter, with persistent pyrexia and progression into oligoanuric stage 3 AKI. Abdominal ultrasound requested for mildly elevated transaminases identified splenomegaly of 18 cm. This finding was confirmed by CT imaging of his chest, abdomen and pelvis (Fig. 1), demonstrating also para-aortic (10 mm) and porta hepatis (16 mm) lymph nodes.

**Fig. 2:**
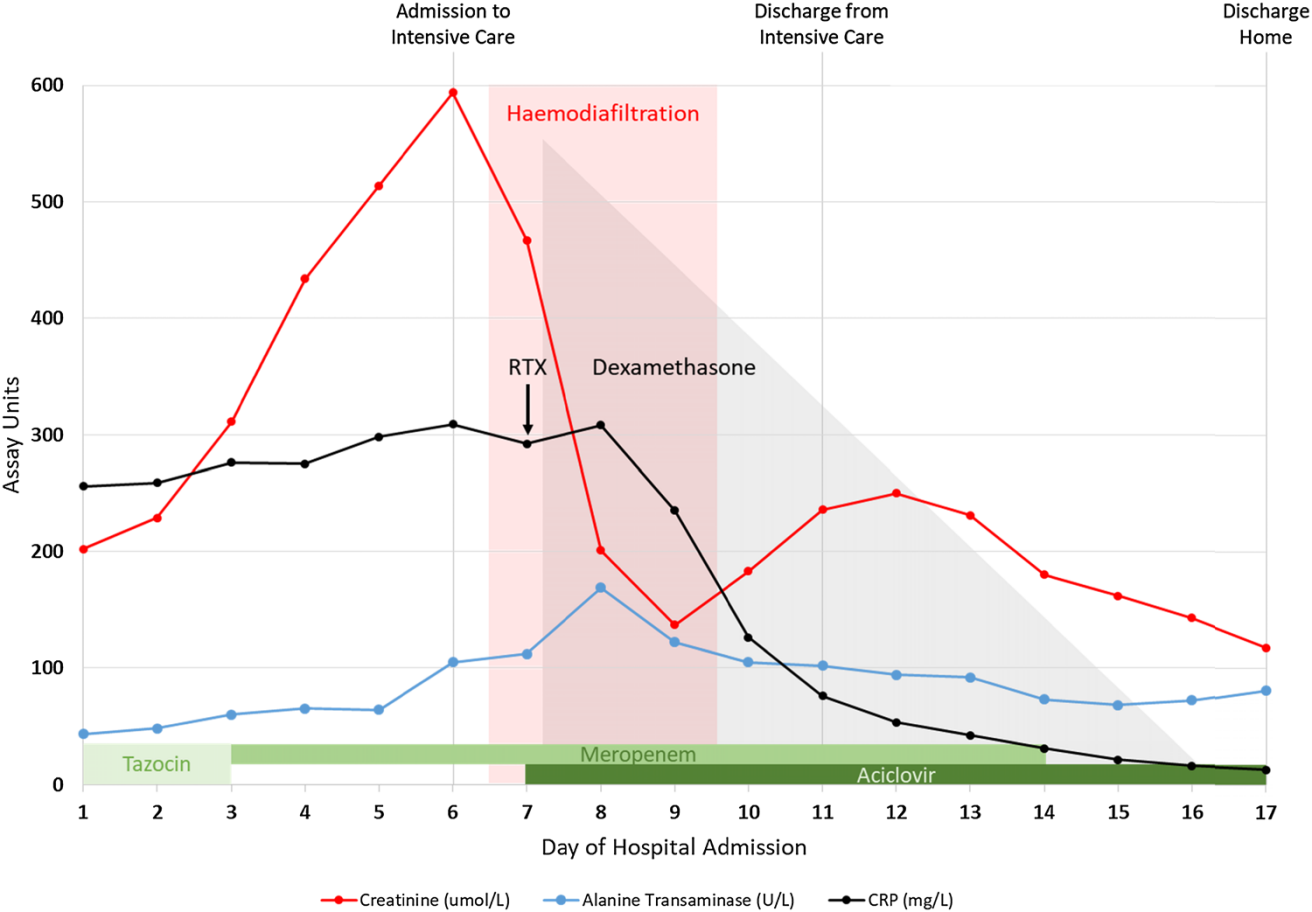
Dynamics of key laboratory markers in response to treatment. Patient’s daily serum creatinine (red line), alanine transaminase (blue line) and CRP (black line) measurements during his hospital admission are shown. Periods of renal replacement (red shading), dexamethasone (grey shading) and rituximab therapy (RTX; black arrow) with antimicrobial coverage (green shading) are indicated

On day 7, our patient became acutely encephalopathic without focal neurological signs, and laboratory tests showed a worsening inflammatory response, elevated transaminases and coagulopathy (Fig. 2). He was transferred to intensive care unit for close monitoring and haemodiafiltration, and his antibiotics were escalated to high-dose meropenem and acyclovir to cover for possible CNS infection. His respiratory function was remarkably well preserved throughout this deterioration.

Repeated sputum, urine and blood cultures from our patient yielded no growth. A lumbar puncture was undertaken but the cerebrospinal fluid biochemistry was unremarkable and analysis was reassuringly negative both for bacterial and viral infection. At this point, our working diagnosis shifted towards EBV reactivation triggered by COVID-19, causing post-transplant lymphoproliferative disorder (PTLD) and/or secondary haemophagocytic lymphohistocytosis (HLH).

Our patient’s precipitous decline unfortunately precluded planned tissue sampling and histological confirmation of either PTLD or HLH prior to treatment. However, since these were the most likely diagnoses and our patient was clinically deteriorating, he received a single dose of rituximab (375 mg/m^2^) together with a 10-day course of IV dexamethasone (dose was gradually tapered down every three days in the following steps: 6 mg twice daily, 4 mg twice daily, 4 mg daily, 2 mg daily) despite ongoing SARS-CoV-2 PCR positivity.

A rapid clinical improvement was achieved with a fall in serum pro-inflammatory cytokines within three days (Table 1). After 17 days of hospitalisation, our patient was discharged with resolved AKI and a CRP of 13 mg/L. Repeated CT scan post-discharge showed reduction of intra-abdominal lymphadenopathy and splenomegaly (Fig. 1B) and serum EBV PCR load of only 50,000 copies/mL.

## Discussion

For the first time, we report a case of a rapid clinical recovery following rituximab and dexamethasone in a critically ill kidney transplant patient with likely COVID-19-driven EBV reactivation and features of secondary HLH. Secondary HLH is a rare but well-established complication of renal transplantation, triggered mostly by infections (in particular, viral such as EBV or CMV) or malignancy [1]. This life-threatening hyper-inflammatory condition is characterised by fever, (hepato) splenomegaly and cytopenias due to unregulated activation of cytotoxic T cells, NK cells and macrophages. Although our patient did not have a bone marrow biopsy, his clinical presentation alone met modified HLH-2009 criteria [2, 3].

Our patient was positive for both SARS-CoV-2 and EBV infection. There is growing evidence from non-transplant patient cohorts that this dual positivity may not be coincidental and could explain, at least in part, the differences in severity of COVID-19 in some patients [4]. Recent case series from Lehner et al. showed that critically ill COVID-19 patients had a higher prevalence of EBV viraemia (78%) than non-COVID-19 controls (29%, *p* = 0.04). While EBV viraemia correlated with COVID-19 patients’ serum IL-6 level, this was not the case in controls (*p* = 0.006) [5]. Similarly, Chen et al. found 55% of 67 COVID-19 patients in Wuhan seropositive for EBV viral capsid antigen (VCA) IgM antibody [6]. Dual-positive patients had significantly higher prevalence of fever, higher CRP and aspartate aminotransferase levels and required more corticosteroids for clinical stabilisation [6]. In a cohort of non-critically ill COVID-19 patients, Gold et al. found a significantly higher prevalence of EBV reactivation in subjects experiencing “long COVID” (i.e. long-term symptoms following a resolution of acute illness) [7]. These studies indicate the potential of COVID-19 to trigger EBV reactivation and lead to worse clinical picture, although the exact (likely immune-mediated) mechanism of this SARS-CoV-2/EBV interaction remains to be characterised. It is important to note that our patient’s supratherapeutic tacrolimus level on admission could have been also another contributing factor driving his EBV reactivation [8].

Although the hyper-inflammatory response typical for severe COVID-19 has features similar to classical secondary HLH, it is rarely associated with (hepato)splenomegaly and haemophagocytosis and almost always involves lungs [9, 10]. Indeed, attempts to adopt HScore (originally designed for diagnosis of HLH) to risk-stratify COVID-19 patients were not successful, further supporting the differences between these two clinical entities [11]. Since our patient did not have any significant pulmonary involvement, we hypothesise that his secondary HLH was driven predominantly by EBV reactivation (primary EBV infection was unlikely given pre-transplant EBV IgG positivity), although the synergistic contribution of SARS-CoV-2 infection could not be excluded.

Once the diagnosis is established, HLH must be treated promptly because of usually rapid clinical deterioration and an otherwise high mortality rate [12]. Current treatment recommendations (HLH-94 and HLH-2004) with dexamethasone, etoposide and cyclosporin are largely based on paediatric protocols for primary (genetic) forms of HLH, and may be suboptimal for the heterogeneous secondary HLH in adults [2]. For example rituximab, an anti-CD20 depleting monoclonal antibody, is often added for EBV-triggered HLH to reduce the virus reservoir in B cells [13]. Treatment of EBV-HLH in a transplant recipient, however, remains a conundrum [14], particularly when presented with a co-infection.

Kidney transplant patients are at high risk of severe COVID-19 infection, due to their immunosuppression, underlying chronic kidney disease and high prevalence of diabetes and hypertension. As such, they appear to have significantly higher early mortality with COVID-19 when compared with the general population [15, 16]. During our initial management of this case, we opted to minimise our patient’s maintenance immunosuppression to enable antiviral immune responses against EBV and SARS-CoV-2. We recognise, however, that reductions in immunosuppression have been suggested to fuel unregulated immune activation and HLH [14]. Moreover, Willicombe et al. recently highlighted the evidence for potential direct antiviral properties of CNIs in COVID-19 [17].

A progressive deterioration of HLH indices (e.g. serum ferritin) in our patient triggered an initiation of a modified, etoposide-free treatment protocol with dexamethasone and rituximab. Although rituximab is generally accepted for treatment of EBV-HLH, its safety in active COVID-19 has not been established, with only one (non-transplant) case report published to date [18]. Mehta et al. speculated that whilst rituximab might be risky at the beginning of SARS-CoV-2 infection by interfering with development of protective antibodies and viral clearance as recently showed by Avouvac et al. [19], it might prove beneficial later (when seropositive) by reducing COVID-19-associated (HLH-like) hyper-inflammation [20]. However, robust data to support such claim are lacking. Indeed, our patient tested negative for anti-SARS-Cov-2 antibodies both shortly after discharge and also three months later. The anti-inflammatory effect of B-cell depletion in late COVID-19 could be linked to antibody-independent pro-inflammatory functions of B cells and formation of immune complexes, but also to their role as EBV hosts [20].

Since EBV-HLH seemed the most likely driver of our patient’s fast clinical deterioration, we concluded that the benefits of rituximab overweighed the risks. Moreover, it was plausible that some anti-SARS-CoV-2 adaptive immunity had already been established two weeks after symptoms onset. He was given only a single rituximab dose (375 mg/m^2^), which was unlikely to trigger a complete B-cell depletion. Following this, our patient’s clinical picture improved with stable respiratory function, suggesting no COVID-19 deterioration. Although it is impossible to disentangle the life-saving treatment effect of rituximab from dexamethasone, this case demonstrates that rituximab use in a rapidly deteriorating SARS-CoV-2-positive kidney transplant patient with probable EBV-HLH and no severe respiratory compromise was an option despite his SARS-CoV-2 seronegativity. Hopefully, future studies will explore soon the expected changes in the incidence of EBV + HLH or PTLD in transplant recipients during COVID-19 pandemic and rituximab safety when treating patients with SARS-Cov-2 and HLH/PTLD. Hopefully, the advent of anti-Sars-Cov-2 therapeutic antibodies and other targeted treatment will substantially mitigate the infection-associated risk of rituximab treatment in cases like ours.

## Declarations

### Human and/or animal participants

This article does not contain any studies with human participants or animals performed by any of the authors.
